# Editorial: Discerning active TB from latent infection

**DOI:** 10.3389/ftubr.2025.1623373

**Published:** 2025-06-26

**Authors:** Pere-Joan Cardona, Synne Jenum, Novel N. Chegou

**Affiliations:** ^1^Microbiology Department, Northern Metropolitan Clinical Laboratory, Hospital Universitari “Germans Trias i Pujol”, Badalona, Spain; ^2^Experimental Tuberculosis Unit, Germans Trias i Pujol Research Institute (IGTP), Badalona, Spain; ^3^Genetics and Microbiology Department, Autonomous University of Barcelona, Cerdanyola del Vallès, Catalonia, Spain; ^4^Department of Infectious Diseases, Oslo University Hospital, Oslo, Norway; ^5^South African Medical Research Council Centre for Tuberculosis Research, Division of Immunology, Faculty of Medicine and Health Sciences, Stellenbosch University, Cape Town, South Africa

**Keywords:** TB spectrum, lung microenvironment, precision prevention, tuberculosis, upper lobes, blood biosignatures, latent tuberculosis infection

## Introduction

**Figure 1:**
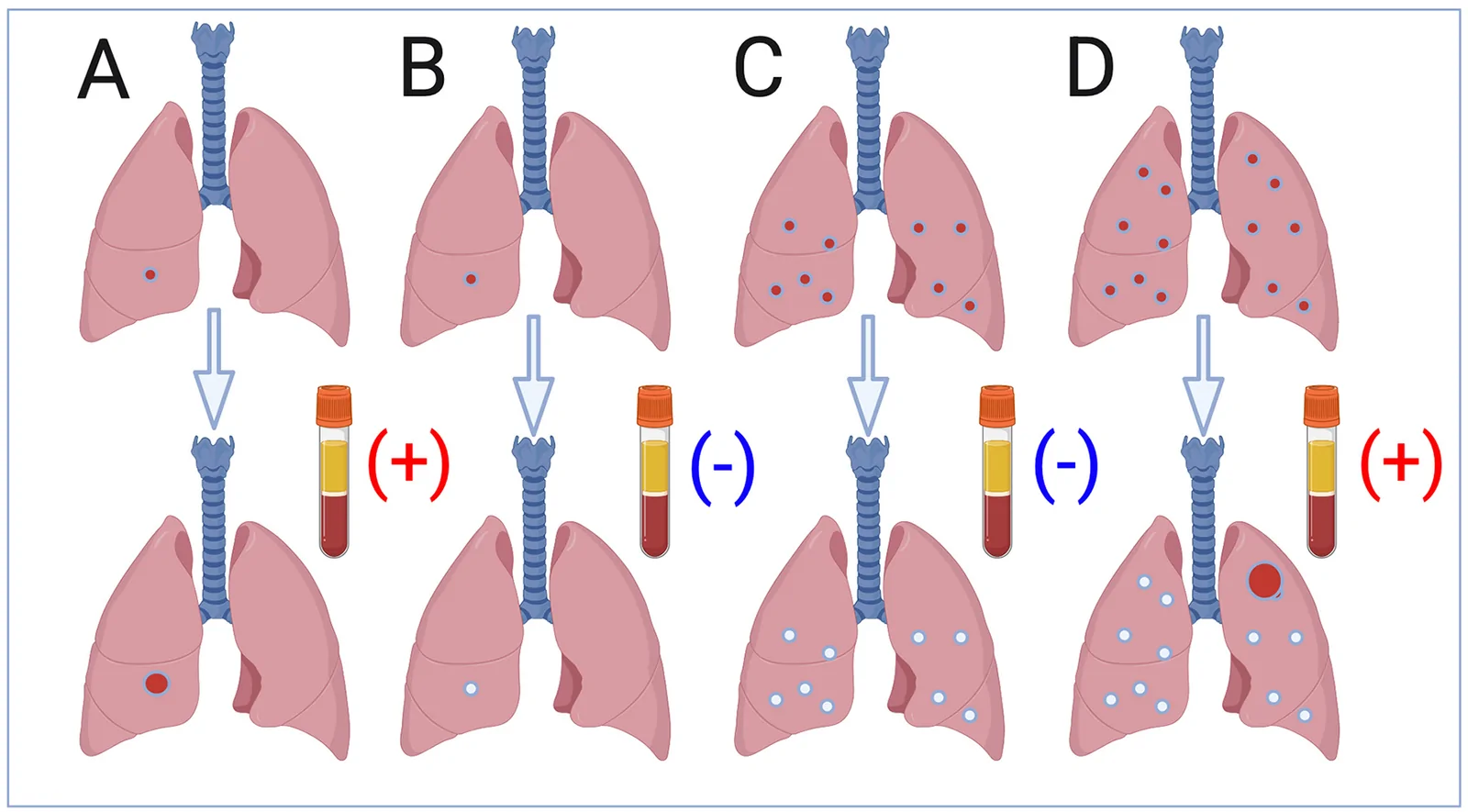
The lung microenvironment: where progression begins. The current challenge in identifying a predictive biosignature for tuberculosis (TB) progression in individuals infected with *Mycobacterium tuberculosis*, represented with the tube with a positive outcome. Pediatric TB presenting as a Ghon nodule **(A)**; non-progressive pediatric infection **(B)**; non-progressive adult infection with multiple infectious foci in the basal lobes **(C)**; TB in adults following progression of a single infectious focus in the upper lobe **(D)** (created by BioRender.com).

The progression from *Mycobacterium tuberculosis* (Mtb) infection to active tuberculosis (TB) is one of the most elusive processes in infectious diseases. Despite extensive global control efforts, the unpredictable nature of this transition continues to fuel TB's persistence as a leading cause of mortality. Current research is converging on a key idea: TB progression is not a binary event but a multifaceted process, shaped by immune responses, lung anatomy and physiology, pathogen virulence and immune evasion strategies, host comorbidities, and environmental pressures like pollution and smoking? This editorial reviews the contributions made to on the topic “Discerning active TB from latent infection” which has been summarized in Figure 1.

## A shift in paradigm: the spectrum of TB infection

Traditionally, TB has been described as existing in two distinct states—latent and active. However, this binary view fails to capture the complex dynamics observed in patients and animal models. A more accurate “TB spectrum” model has been proposed, in which bacilli may cyclically grow, remain dormant, heal naturally, or reinfect the host (Cardona) (1, 2). The fluctuating balance within a single lesion and in the surrounding microenvironment is influenced by local anatomical and immunological variables. These factors determine whether lesions progress or remain contained, but varies even within the lung of the same host thus potentially result in healing of some lesions but progression in others.

This model explains why conventional diagnostic approaches often fall short. Relying on biosignatures in blood misses has limited value, as reviewed recently (3). Finally, localized changes occurring mainly in the upper lobes of the lungs—the true arena of TB evolution.

## The lung microenvironment: where progression begins

One of the most ground-breaking insights into TB progression comes from the comparison of systemic and local immune responses. Ahimbisibwe et al. have demonstrated that tissue-specific immune signatures, particularly in the lungs, provide more reliable indicators of latent TB reactivation risk than peripheral blood markers. Their study has found that individuals with positive IGRA tests who later progressed to active TB had significantly more CD4+ tissue-resident memory T cells and KLRG1+ terminally differentiated CD4 T cells in lung tissue. These features, absent in blood, suggest persistent immune engagement at the infection site—potentially due to intermittent bacillary activity. On the other hand KLRG1+ might mark immune exhaustion, thus potentially indicating early progression toward active TB.

This localized immune “alertness” supports the claim that progression is largely a pulmonary event (4). The anatomical structure of the upper lobes—characterized by reduced perfusion, weaker lymphatic drainage, and diminished fibroblast responsiveness—creates an environment prone to lesion expansion. When the balance between containment and inflammation is disrupted, progression is more likely.

## Molecular signals: predicting progression before symptoms

At the molecular level, early progression from latent to active TB can now be detected through subtle shifts in gene expression in the host? Daniel et al. have identified a panel of seven microRNAs (miRNAs) whose altered expression predicted TB progression with striking accuracy. These miRNAs regulate processes such as apoptosis, immune suppression, and autophagy—each of which plays a role in allowing Mtb to persist and evade immune control.

Notably, these biomarkers were extracted from QuantiFERON supernatants, suggesting they reflect TB-specific immune activity. Their predictive power met the WHO's benchmarks, but more importantly, they highlight the potential of a blood biosignature to predict disease well before clinical signs emerge. These findings bridge systemic and cellular responses, bringing us closer to a reliable prognostic tool.

## Functional immune profiling: the cytokine code

Immunological function—not just presence—also holds vital clues. Zhang et al. have evaluated a triple-color FluoroSpot assay that simultaneously measured IFN-γ, TNF-α, and IL-2 secretion from antigen-stimulated T cells. Their results have revealed that patients with active TB showed elevated IFN-γ and TNF-α, while IL-2 dominated in those with latent infection. These patterns are consistent with a shift from regulatory to inflammatory cytokine responses during progression—a phenomenon that may trigger lesion enlargement and extracellular bacillary growth.

Such tools offer diagnostic resolution beyond static tests like IGRA, helping clinicians understand not only whether someone is infected, but where they lie on the path toward TB progression.

## Comorbidities and radiological predictors of risk

Progression is also influenced by host vulnerability. Lu et al. have developed a predictive model for poor treatment outcomes during the intensive phase of TB therapy. While the model focused on active cases, its findings shed light on the risk factors that likely play a role in progression. These included diabetes, tracheobronchial TB, lung cavities, elevated CRP, and anemia—each a marker of weakened systemic or local immune resilience.

Importantly, these authors have emphasized the use of CT imaging to monitor lesion evolution, especially when sputum tests are negative (Lu et al.). This echoes the call to focus on lesion location and morphology rather than just bacillary presence (4). The upper lobes, for instance, are mechanically predisposed to allow lesion expansion due to their structural characteristics and gravitational stress.

## Diagnostics in transition: adapting to the complexity of TB

As our understanding of progression evolves, so too must the tools we use to diagnose and monitor TB. Mugenyi et al. have reviewed the spectrum of emerging diagnostic technologies, from rapid molecular platforms like GeneXpert Ultra and LAMP to host-response assays like immuno-PCR and aptamer-based antigen detection. While many of these tools are still under evaluation, the trend is clear: future diagnostics must be integrated, affordable, and capable of detecting both pathogen presence and host immune responses.

For populations most at risk—children, HIV co-infected individuals, and those with subclinical disease—non-invasive and highly sensitive tools are crucial. Stool testing for pediatric TB and urine-based LAM testing in HIV patients represent important steps in this direction. Yet, affordability and accessibility remain major barriers (Mugenyi et al.).

## The evolutionary context of TB progression

Finally, understanding TB progression requires us to consider evolutionary and environmental contexts. It has been suggested that Mtb was once a symbiotic organism, enhancing human immune responses and rarely causing lethal disease (4). Changes in lifestyle—urbanization, industrialization, and chronic stress—may have disrupted this balance. Today, men are consistently more affected by TB, possibly due to stress-induced cortisol elevation. These social determinants, along with comorbidities like undernutrition, alcohol abuse, smoking, living with HIV and diabetes mellitus appear to be active players in the progression equation.

TB is not just a biological challenge; it is also a product of the environments and systems we live in.

## Toward precision prevention: a unified strategy

All these studies collectively argue for a paradigm shift. Progression from infection to active TB is not an inevitable event—it is a complex, modifiable process. By recognizing the role of immune exhaustion, structural lung vulnerabilities, miRNA dysregulation, cytokine profiles, and social context, we can build smarter diagnostic tools and tailored interventions.

Moving forward, the most effective strategies will be those that integrate clinical, molecular, immunological, and imaging data to generate individualized risk profiles. This is the future of TB control: predictive, personalized, and precise.
